# Report of the Diseases of Edinburgh for May, 1810

**Published:** 1810-08

**Authors:** John Roberton


### *Report of the Diseases of Edinburgh for May,* 1810.

By John Roberton, M. D.

Before the commencement of the month, the weather had become dry, and it continued so during about one-third of it. Till this time, when we had some gentle falls of rain, vegetation was so parched as to be almost completely arrested in its progress. From this period till the termination of the month, we had frequent falls of rain, and some thunder storms.

The barometer, during the early part of the month, stood high, and even during the succeeding rains, it did not fall considerably.

The thermometer stood in general from 55 to 70, and, once or twice, it rose even several degrees higher.

During this period, there scarcely seems to have been any one complaint of such a prevalent or troublesome nature as to require any very particular description. There were various slight affections, such as catarrh, cynanche tonsillaris, &c.; also some cases of fever, which are indeed, never absent from this place; some few remaining cases of chin-cough, and a few of hydrocephalus internus.

This scarcity of disease among us seems to be owing partly partly to the nature of the season, and partly to the great number of families which have retired into the country; at which times they carry with them not only the healthy but also the sick.

